# An Improved Gray Neural Network Method to Optimize Spatial and Temporal Characteristics Analysis of Land-Use Change

**DOI:** 10.1155/2022/2699031

**Published:** 2022-08-11

**Authors:** Yang Yang, Wei Wang, Jiajun Qiao, Ershen Zhang

**Affiliations:** ^1^The College of Geography and Environmental Science, Henan University, Kaifeng 475004, China; ^2^School of Culture Industry and Tourism Management, Henan University, Kaifeng 475004, China

## Abstract

In this article, the principles of the gray model and BP neural network model are analyzed, and the characteristics of land-use change and spatial and temporal distribution are studied in-depth, and at the same time, to explore the influence of land-use change on ESV, the relationship between the two is analyzed using gray correlation degree, and a mathematical model is constructed to maximize the benefits of the regional system, coupling economic and ecological benefits, combined with Geo SOS-FLUS model to achieve the optimization of land use. This article constructs a combined prediction model of a gray neural network. The gray differential equation parameters correspond to the weights and thresholds of the neural network, and the optimized parameters are determined by training the neural network to make it stable. Then the training results of the BP neural network are fitted with the results obtained from the gray GM (1.1) model. Finally, the prediction results of the three models, gray GM (1.1), BP God Meridian, and gray neural network model, are compared and analyzed. The global spatial autocorrelation and local spatial aggregation patterns of regional soil erosion and its erosion factors are analyzed using the Exploratory Spatial Data Analysis (ESDA) method in spatial measurement theory.

## 1. Introduction

Land change is a visual representation of the changes in natural conditions and the extent of human activities. An in-depth understanding of land change's spatial and temporal characteristics and its influencing factors provides a basis for revealing global ecosystem changes and provides theoretical values and practical guidance for improving the regional environment and rational allocation of land resources. Land-use cover change is based on natural environmental conditions. The natural environment conditions will differ significantly in different regions, so the land-use change will also be other. There is a close spatial and temporal correlation between human drivers and land-use change [1]. The various land cover change phenomena generated in a region reflect different human factors' direct and indirect effects. Studies have shown that among the drivers of land-use change, the influence of socio-human factors is more prominent and relatively active. In contrast, the result of natural elements is more permanent and robust. In short time scales, the power of human activities plays a more prominent role and is usually in a dominant position. In addition, land use is a direct manifestation of decision-making at multiple scales and dimensions, and natural and human drivers have different degrees of influence at different scales of time, space, and participants. Even the same driver plays different roles in other regions.

Geographic Information System (GIS) spatial analysis technology is the use of the relevant spatial analysis tools with the help of computers running GIS-related software platforms to operate and analyze geographic data to obtain and transmit the geographic location, distribution, morphology, spatial relationships, and other spatial information that cannot be extracted intuitively, which provides a practical technical platform, land-use area delineation, etc. It has been widely used in land, water conservancy, aerospace, mapping, transportation, meteorology, etc. It has been commonly used in land, water, space, mapping, transportation, meteorology, etc. Artificial intelligence (AI) technology has been integrated into various fields of socioeconomic development, and AI technology makes it possible to automatically identify and capture implicit, disordered, and many data features [2]. As a significant branch of artificial neural network algorithm, Self-organizing Feature Mapping (SOFM) neural network is self-organizing clustering of the target dataset with its internal mutual learning and competition mechanism. The clustering results are spatially continuous and similar in attributes, suitable for classifying uncategorized multidimensional data sets.

To carry out scientific zoning of the spatial and temporal configuration of land remediation in the context of spatial reconstruction of “three lives,” it is necessary to consider multiple factors such as the ecological risk of land remediation, the urgency of land remediation, and appropriateness of land remediation [3]. Modern information technology such as GIS spatial analysis technology and artificial intelligence algorithms such as SOFM neural networks can simultaneously consider multilevel, multiobjective, multidimensional, and many data indicators. It can reduce the human subjective arbitrariness in the calculation process to a large extent, which has advantages in spatial and temporal land remediation zoning. It is superior in land remediation spatial and temporal configuration zoning. The research method of GIS spatial analysis technology SOFM neural network for spatial and temporal configuration zoning of land remediation can consider the geographic location and spatial attributes of land remediation and improve the objectivity and implement ability of zoning results, which will help the traditional localized land remediation of “digging and burying” to “field, water, road, forest, village, and enterprise.” It will help to change the direction of “field, water, road, forest, village, and enterprise” to comprehensive natural resource remediation, which can reconstruct the “three living” spaces of the countryside, optimize the “three living” functions, and carry out the rural revitalization strategy in the new era [4]. It can provide theoretical and methodological support for deepening the rural revitalization strategy and the construction of agricultural ecological civilization and create a unique pattern of “land improvement + multiple elements” that integrates ecological, urban-rural integration, refinement, and comprehensiveness and human culture land improvement.

## 2. Related Works

LUCC is the essential carrier of the production and life of human society. The study of LUCC is beneficial to the degree of coordination between the regional human and natural environment because human activities most directly affect land use, such as food production and extraction of water resources. At the same time, land-use changes also have the most direct impact on humans; if human beings exploit the land unreasonably, the balance between the two can easily be broken, leading to the loss of control of ecosystem and energy circulation, which in turn will cause turbulence to global species and crustal surface structure, and many problems such as extreme weather, soil erosion, and biodiversity reduction [5]. Currently, it is widely used in predictive simulation studies. Certain shortcomings of the regression analysis model are mainly that the model structure and parameters must be artificially determined before data analysis is conducted, so the method is somewhat subjective. It carries uncertainties in reflecting the intrinsic laws of the data. Bui selected several drivers of economic, social, and spatial distances [6]. They applied a logistic regression model to explore the drivers affecting the changes in arable land, forest land, and grassland in the mining area at different time intervals. Kang et al. constructed a driving force model of land-use change in the city and used logistic regression analysis to quantitatively analyze the relationship between factors affecting land-use change and spatial drivers, and to simulate and predict the spatial pattern of future construction land, and concluded that logistic regression model is an effective method and approach for land-use change analysis and simulation prediction [7]. The system dynamics model, which is more applicable in analyzing the internal structure and quantity prediction, can reflect the complex behavior of the land system from a macroscopic perspective and is better applied in the scenario simulation study of land-use change. Still, the model lacks spatial analysis tools, so it is difficult to realize the simulation of land-use spatial distribution [8].

The gray prediction model applies the theory of mathematical statistics to time series and makes forecasts based on it. The model cannot summarize the combined effects of multiple influencing factors, so many researchers believe that the gray prediction model is more applicable when making short-term predictions of land-use change; if long-term predictions are made, the accuracy will be reduced due to the interference of various factors with each other. Pan et al. analyzed the future land use of the region through the gray prediction model, and the prediction results were compared with the results predicted from statistical data to verify that the gray prediction model is consistent with the purpose of the study [9]. The famous scholar Professor Kouadri et al. proposed the gray system theory, which can solve the analysis and prediction problems of small sample and poor information systems [10]. Its basic idea is to combine small pieces of known data related to time by mathematical methods to form white modules and then predict unknown gray modules. And the process of mining surface deformation data collection will be affected by various uncertain factors, and the degree of influence is not certain; not easy to describe with a definite function model; these characteristics are precisely in line with the research problems of the gray model. So many scholars have applied the gray theory model to predict surface deformation data in mining areas and have achieved some achievements. Casazza et al. established a gray Verhulst model to simulate and study the surface movement process [11]. Zhang et al. showed a non-equally spaced GM (1, 1) model to predict the surface subsidence of mine mining for the characteristics of non-equally spaced mining subsidence monitoring data [12]. Alam et al. established a gray DGM model to predict ground subsidence based on observation data [13]. Hurtt et al. showed an equidimensional new interest gray model to predict the surface subsidence value in mining areas; all achieved better results and proved the feasibility of the gray model in surface deformation prediction [14].

A neural network is a modeling method proposed by Han and Zhu based on an analysis and summary of the fundamental properties of neurons [15]. The BP algorithm is one of the most applied algorithms for the neural network model, which is trained by treating the monitoring data as samples of the model and improving the approximation accuracy in continuous learning, and the neural network model can simulate the displacement of the monitoring location after determining the network model. Powers and Jetz established a neural network prediction model for surface subsidence in coal mines [16]. Borrelli et al. used the improved neural network prediction method to predict mining subsidence and surface movement under thick loose layers [17]. Fonseca et al. built a BP neural network model with pre-processed observation data to predict surface subsidence in mining areas [18]. Sasmito et al. established a time-series BP network model by example and trained and tested the network with existing observation data [19]. Davison et al. used a genetic algorithm to optimize the initial weights of the BP neural network for the regional ground settlement problem [20]. They established a ground settlement prediction model, which overcame the disadvantages of slow convergence and easy falling into local minima of the BP neural network model. Chen et al. established a residual settlement prediction model for the old mining area by using the existing partial observation data after the maximum subsidence velocity of the observatory and the BP algorithm of L-M [21].

## 3. Improved Gray Neural Network Model Construction

The objective of gray system theory is an uncertain system where “some information is known, and some information is unknown.” By analyzing the changing pattern of the gray system and its characteristics, valuable or regular information can be extracted from it to achieve the purpose of accurate description and effective control of system behavior and evolution law [22]. The shade of color usually describes the degree of information perception in a gray system; the “white” expression knows part of the information. The gray system is a system of incomplete information. Gray system modeling is to use fewer available parts of the data through the cumulative generation of transformation of known data, the chaotic and disorderly data series into monotonic growth series, which strengthens the data regularity, establishes the differential equation coinciding with the system change, and predicts the unknown part of the information, to understand the trend and law of data change. The gray system model requires the modeled data to have quasi-exponential regulations, which require the discrete data *x*^0^*k* to have smoothness to ensure that the modeled series has exponential laws. Thus, the modeled series must be smoothly determined before modeling.

(1) $$\begin{array}{c} P_{k}=\underset{i=1}{\sum }x^{0}\left(i\right)-x^{\left(0\right)}k. \end{array}$$

The gray system model is to establish differential equations for discrete series, and the GM model is to develop first-order differential equations with the expressions:

(2) $$\begin{array}{c} u_{k}=\sum \frac{\text{d}x^{1}}{\sqrt{\text{d}t-ax}}+ax, \end{array}$$

where a and *u* are parameters to be determined, *a* is called the development coefficient of the model, *u* is called the amount of gray action of the model, and *x*^0^ is the sequence generated by one accumulation of the original series *x*^1^. The actual data of the gray model modeling *x*^0^*k*=(*x*^0^(1), *x*^0^(2),…*x*^0^(*n*)) is a nonnegative sequence, and after one expansion to develop *x*^1^*k*=(*x*^1^(1), *x*^1^(2),…*x*^1^(*n*)), then its accumulation formula is:

(3) $$\begin{array}{c} x\left(k\right)=\underset{i=1}{\sum }\frac{\sqrt{x^{0}}}{i}. \end{array}$$

**Figure 1:**
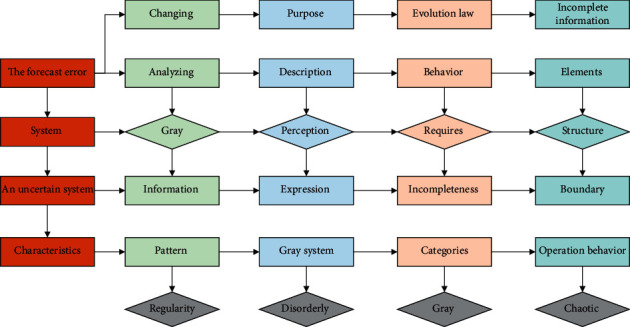
Flow chart of improved gray GM (1, 1) model prediction.

The improved gray model is mainly constructed by combining the gray and ARIMA models. The principle is first to forecast freight turnover using the model, calculate the forecast error, make the mistake ARIMA forecast model, and add the forecast values of the two models to arrive at the forecast value of freight turnover. The flow chart of improved gray GM (1, 1) model forecasting is shown in Figure 1. The improved gray model: (1) does not require many samples; (2) the samples do not need to be regularly distributed; (3) the computational effort is small; (4) the results of quantitative analysis will not be inconsistent with the results of qualitative analysis; (5) it can be used for recent, short-term, and medium-to long-term forecasting; and (6) the accuracy of gray forecasting is high.

The specific modeling steps of the improved gray GM (1, 1) based logistics demand forecasting model are as follows.

(1) For the sample *x*^0^*k*=(*x*^0^(1), *x*^0^(2),…*x*^0^(*n*)), a prediction model was developed to simulate the original data series to obtain the fitted and gray prediction series *x*^1^.
(2) Find the simulation error series *E*_1_^0^ for the fitted series *x*^0^.
  (4) $$\begin{array}{c} E_{1}^{0}=\frac{x^{0}n+x^{1}n}{\sqrt{e_{1}-e_{2}}}. \end{array}$$
(3) Find the minimum value *E*_(1min)_^0^ in *E*_1_^0^ and correct the simulation error sequence *E*_1_^0^ to obtain the corrected error sequence *E*_2_^0^ The error correction formula is:
  (5) $$\begin{array}{c} E_{2}^{0}=\sum E_{1}n-\left|E_{\left(1\min\right)}^{0}\right|. \end{array}$$
(4) Calculate the smoothness of the error series *E*_2_^0^ and do an ADF test on the error series *E*_2_^0^ to determine the difference order *d*.
(5) Establish an ARIMA model and use EViews software to obtain autocorrelation and bias correlation plots, identify and rank the model according to the correlation characteristics, and then find the optimal model corresponding to the minimum value of the one-step error of the model *p*, *q* by the AIC criterion.
(6) Reduce the corrected error prediction sequence to the prediction sequence *E*_1_^0^ of the error sequence *E*_2_^0^; the reduction formula is:
  (6) $$\begin{array}{c} E_{1}^{0}=\frac{E_{2}^{0}\left(n-1\right)}{E_{\left(1\min\right)}^{0}}. \end{array}$$
(7) The ARIMA model prediction results are tested for white noise, and if they do not pass, we go back to step (5) until the optimal model is obtained.
(8) The predicted result series of the gray model *x*^0^ is added with the expected result series of the modified Arima model *E*_1_^0^ to obtain the desired data series of the combined model *Y*^0^.
  (7) $$\begin{array}{c} y\left(k\right)=\sum \frac{E_{1}^{0}\left(k-1\right)}{x^{0}-1}. \end{array}$$

**Figure 2:**
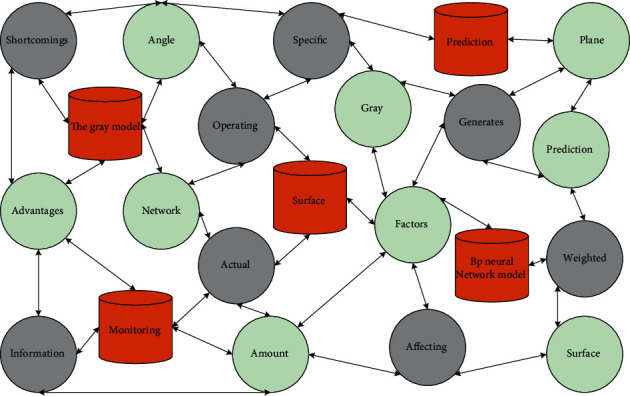
Combined form of improved gray neural network.

Since the non-equally spaced gray model generates the gray prediction plane, the result obtained is the gray interval of the predicted value. However, specific prediction data are sometimes needed, so the expected value's gray break must be weighted. The BP neural network model is established with various factors affecting the land surface deformation data, that is, dip angle, depth ratio, and operating surface advancement speed as sinkage amount at the monitoring point as the output information of the neural network. BP neural network models and traditional neural network models are used for different purposes. Feedforward neural networks: the main applications include perceptron networks, BP networks, and RBF networks. The combined model can give full play to the advantages, avoid the shortcomings of the individual prediction model, and get the results with higher accuracy. Its collaborative form is shown in Figure 2.

From the combined state of the gray model and BP neural network model, it is known that the gray neural network model belongs to the tandem type connection, so according to the modeling principle of the tandem combinatorial model, its modeling steps are.

1. Modeling the original series by a non-equally spaced model, calculating the boundary function of the gray prediction plane, and deriving the gray interval *P*.
2. Taking the part of the gray interval *P* corresponding to the known data sequence, the actual value *T* of the known data sequence as the BP neural network output, and setting the network structure, initial weights, and thresholds.
3. The sequence data related to the predicted values in the gray interval *P* is used as the prediction input of the network, and the simulation is calculated to obtain the network output values.

## 4. Spatial and Temporal Characteristics of Land-Use Change Analysis Model Design

Land-use change is the primary manifestation of human activities that interfere with the natural ecosystem. Land-use change research has become essential for environmental protection, land development and utilization, and management. It is an essential reference for measuring temporal differences in regional land-use changes and predicting future land-use changes [23]. The regional land-use change research mainly uses various land-use change analysis indices to reflect the characteristics of land-use change. The main methods applied in this article are:

(1) Land-use emotional attitude refers to the change in the quantity of a particular land type in the study area within a specific time, which can precisely reflect the drastic degree of change in regional land-use or cover for a specific time, calculated by the formula.
  (8) $$\begin{array}{c} k=\frac{\left(u_{ib}-u_{ia}\right)}{\sqrt{u_{ib}T}}. \end{array}$$
(2) This article calculates the changes and transformation relationships of each land-use type in the district with the help of the land-use transfer matrix, which is calculated as follows: *B* represents the area of the study area; *n* represents the different land-use types; and *i* represents the land-use types at the beginning of the study.
  (9) $$\begin{array}{c} B_{ij}=\left[\begin{array}{cccc} b_{1} & s_{2} & s_{13} & s_{n-1}\\ b_{2} & b_{2} & b_{5} & b_{2-n}\\ b_{3} & b_{4} & b_{6} & b_{3-n}\\ b_{4} & b_{5} & b_{8} & b_{4-n} \end{array}\right]. \end{array}$$

**Figure 3:**
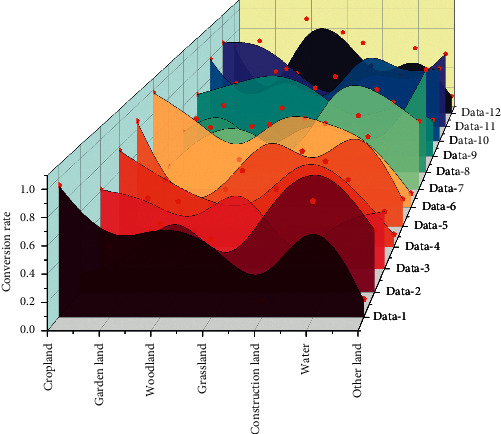
Land use change conversion.

The Markov model can reasonably predict the change in the number of various land types in the study area but cannot calculate the spatial layout of land use; the basic principle of the FLUS model is derived from the meta-cellular automaton (CA) and has made a significant improvement on the general meta-cellular automaton, which can reasonably simulate the land-use change under human activities and natural conditions. The Markov model can predict the quantitative changes of various land types in the study area but cannot calculate the spatial layout of land use. It has been dramatically improved on the general meta-cellular robot, which can also simulate the land-use changes under human activities and natural conditions. The Markov-FLUS composite model constructed by combining Markov chains and beta cellular automata has both the quantitative prediction ability of the Markov model and the power of the FLUS model to deal with the distribution of beta cells in space to process the miscellaneous land-use transformation information. The simulation of the predicted demand of Markov as the driving force of the FLUS model can be well simulated for the long-term land-use situation with sure scientific accuracy [24], which can reflect the overall interconversion between various land-use types in the period and then combine with the module of beta cellular automata in the FLUS model to obtain the quantitative prediction of land use and its distribution in spatial location through evolutionary simulation. When a land type can be converted to another, the corresponding matrix value is set to 1; when conversion to other types of land is not allowed, it is set to 0. The land-use change conversion is shown in Figure 3.

The first law of geography shows that things are interconnected in spatial distribution. The land-use transfer matrix is a quantitative research method that uses spatial analysis to determine the amount and direction of interconversion between land-use types over time. And spatial autocorrelation can detect the spatial dependence between variables through the accumulation of data or spatial interaction and reveal the regional structure pattern of spatial variables. The degree and significance of the global spatial autocorrelation are calculated as follows.

(10) Moran'sI=∑i=1∑j=1wijai+a∑i=1∑j=1wij∑i=1ai−a.

Moran's*I*, the index is an overall statistical indicator that only reflects the degree of difference in the spatial distribution of cropland across the region and does not specify the location of clusters and the type of spatial autocorrelation, which requires local indicators of spatial autocorrelation to explain the interaction between the adjacent units in the local area. This study applied the Hot Analysis spatial analysis tool in Arc GIS for Getis-OrdGi^*∗*^ index analysis. We used the natural breakpoint method to spatially explore cropland distribution's cold and hot spots in each township unit. The data for the detector statistics need to be discrete, and the qualitative data are discrete [25]. They can be detected, realizing a full range of analyses of the influencing factors. The detector can see whether any two factors influence the dependent variable, including a shared influence of the two factors on the dependent variable, strong and weak, positive and negative, linear or nonlinear, etc. When dealing with surface data, *Y* is first uniformly discretized and then superimposed with the distribution of the independent variable to achieve a spatial match between the two variables and facilitate extraction. When dealing with point data, there are two cases, when the point data can represent the total, the calculation can be performed between them, and when the point data cannot describe the whole, the data need to be further corrected and corrected before analysis. The detector has relatively low requirements for data and no assumptions and has been applied by more and more scholars in detecting soil erosion, residential life, urban construction, etc., and has made some progress.

## 5. Analysis of Results

### 5.1. Improved Gray Neural Network Model Construction Analysis

It is found that the inherent defects of the GM (1, 1) model are mainly the mismatch between the swept equations and the gray differential equations in the modeling process and the selection of the initial conditions. Compared with the equation mismatch problem, the initial conditions have less influence on the fitting accuracy. The improvement methods add an initial value correction term or solve the optimal initial value by the least-squares principle [26]. In contrast, the more significant equation mismatch problem can use various correction methods, mainly background value construction improvement, gray differential equation modeling, parameter reconstruction of the whitening equation, and straightforward solution parameter method. Among them, background value construction is the most common model improvement method, based on the difference between the value background value 0.5(*x*^1^*k*+*x*^1^(*k* − 1)) and the corresponding ∫_*k*−1_^*k*^*x*^1^*dt* in the whitening equation and the reconstruction of the background value to match the gray differential and the whitening equation.

**Figure 4:**
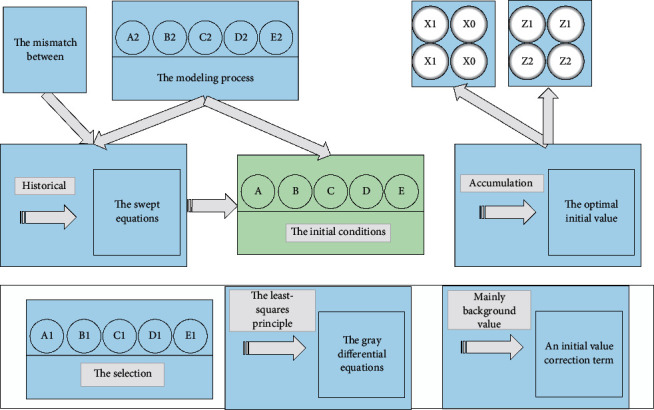
GM (1, 1) model background value optimization process.

In contrast, parameters *a* and *b* are derived from the original data series and the background values, so the reason or not of the background value construction formula directly affects the simulation. Considering the minor sample nature of the measured historical data, a regularized RBF network structure is used to fit the GM (1, 1) model with a single accumulation to generate a sequence *x*^1^. When the formalized RBF network structure is used, the center of the basis function is the sample itself. This article programmatically implements the GM (1, 1) model background value optimization in the MATLAB environment. The regularized RBF neural network is built by the new function. The process of optimizing the background value of the GM (1, 1) model is shown in Figure 4.

**Figure 5:**
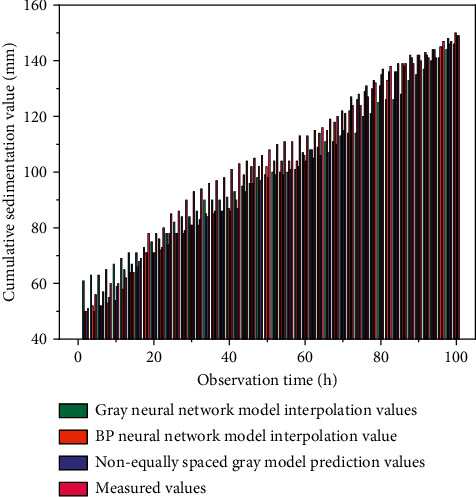
Interpolation results of interrupted deformation data.

The periodic data interpolation based on the non-equally spaced gray model predicts the intermittent surface deformation data of the mine area with the non-equally spaced model. It calculates the non-equally spaced GM (1, 1) model's modeling with available time-series data and derives a series of parameters from the gray model. The fitted values are reduced to the interpolated values of the non-equally spaced model for the intermittent data. Takes the time series corresponding to the original data settlement as its input data, takes the known measured values as the target output, makes a series of settings for the network parameters, trains the network using the L-M algorithm, and takes the time series corresponding to the missing values as the prediction input [27]. The network simulation is calculated to interpolate the missing values in the deformation data. The intermittent data interpolation based on the combinatorial model uses a gray neural network combinatorial model to simulate the periodic deformation data of the land surface for the experiment. According to the modeling principle of the combinatorial model, the non-equally spaced gray model is firstly used to process the known deformation data, that is, the data after transformation processing is generated cumulatively once with the time interval as the weight, and the matrices *B* and *Y* are constructed by taking different background values. The least-squares method is applied to calculate the parameters according to the vectors *a* and derive the two whitened response equations of the gray model. The gray intervals of the predicted values can be obtained by reducing them. Initialize the weights and thresholds of the network, change the training process of the network by adjusting the number of nodes in the hidden layer, and stop the training when the mean square error target function reaches the set training error target value [28]. The output value of the BP neural network is obtained by simulation, and the final prediction value is obtained after its inverse normalization process. The interpolation result of intermittent deformation data is shown in Figure 5.

**Figure 6:**
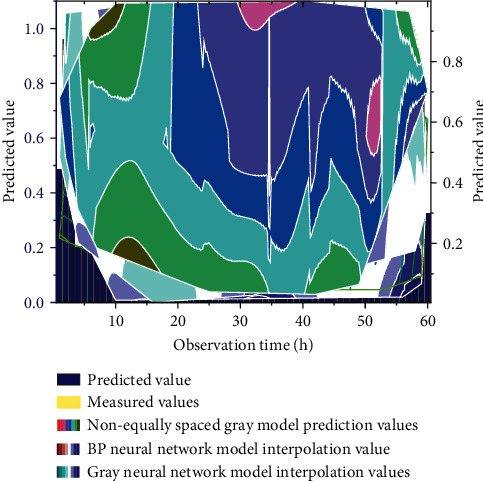
Interpolation results of interrupted deformation data for *k*_1_ + 885.

The average relative error of the non-equally spaced gray model was MRE = 6.72%; the mean relative error was MRE = 6.21% for the BP neural network model, and the mean relative error was MRE = 4.81% for the combined gray neural network model, from the comparison of the interpolation results of the sporadic data, the closeness of the combined gray neural network model to the measured values is significantly higher than that of the gray model and the BP neural network model, which indicates that the combined model has higher accuracy in interpolating the intermittent data. Let the monitoring data of point *k*_1_ + 885 for periods 7–9 be vacant. The prediction model is used to interpolate the data of periods 7–9. The interpolation results are shown in Figure 6.

**Figure 7:**
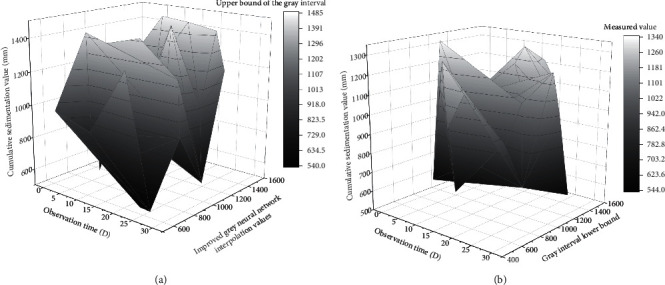
Improved gray neural network prediction results.

Firstly, the function *y*=*x*^1/10^ transforms the original data as a function. Then the transformed data are generated cumulatively once with the time interval as the weight according to the modeling principle of the non-equally spaced GM (1, 1) model, and the matrices *B* and *Y* are constructed by taking different background values; then, the least-squares method is applied to calculate the parameter vectors *a* and *u*, which are substituted into the initial value modified whitening response equation. The modified whitening response equation is used to sink the mine area. The resulting function values are reduced and calculated to obtain two discrete data, and the interval enclosed by these two discrete data is the gray interval of the predicted values [29]. The prediction results of the improved gray neural network are shown in Figure 7.

### 5.2. Realization of Spatial and Temporal Characteristics Analysis of Land-Use Change

**Figure 8:**
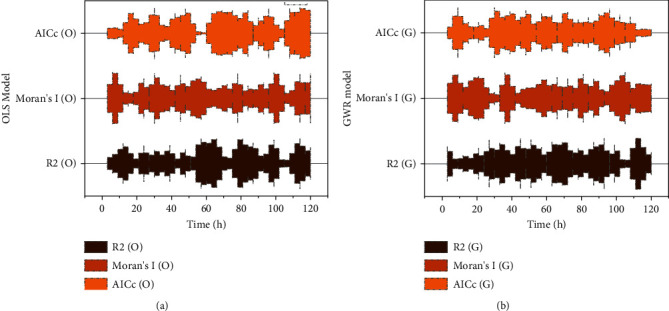
Comparison of OLS model and GWR model.

The adjusted *R*^2^ and AICc can compare the superiority of OLS and GWR models for fitting statistical parameters. Among them, the range of *R*^2^ values is [0, 1], and the more significant value indicates that the model can assess the model performance more accurately. At the same time, AICc is used to reflect the execution effect of the model, and the smaller value indicates that the model has better explanatory power. Meanwhile, this study uses Moran's *I* value to judge whether the residual terms of the independent variables are independent or not on this basis. The more the model can satisfy the assumption of the different distribution of the residual terms. Intuitively, consider OLS as fitting (*x*, *y*) to a rigid shape (e.g., a straight line) of the scatter plot, while GWR allows that shape to oscillate arbitrarily. With the OLS model and GWR model AICc, adjusted *R*^2^ and Moran's *I* value of the residuals, the regression while the OLS model's *R*^2^ value reaches 0.7989, which is 11.21 percentage points higher than the GWR model, that is, the model explains 79.89% of the township cropland change, indicating that the OLS model is more appropriate in terms of fitting effect. Still, its AICc value is 27.15 points higher than the GWR model's 27.15 points. The residual Moran's *I* value is −0.75, that is, its residual term has a negative spatial correlation potential. A comparison of the OLS model and the GWR model is shown in Figure 8.

**Figure 9:**
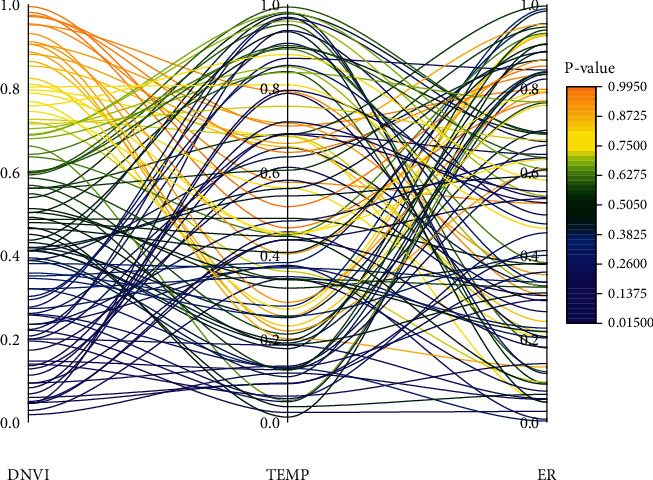
Temporal variation of *P* values for the results of the driving factor index test.

The regression coefficient value of the spatial Durbin panel data model for the SEC factor is −0.386, while the regression coefficient value of the direct effect is −0.387. This phenomenon can be explained by the feedback effect of the spatial model. In the spatial expression of the soil erosion spatial Durbin model, the feedback effect of the soil erosion driver is mainly derived from two parts, one part is the spatial lag term of the explanatory variable of the soil erosion spatial model (*W∗X*) and the other part is the spatial lag term of the explanatory variable of the soil erosion spatial model (*W∗Y*). The feedback effect of the soil erosion control factor is its direct effect. The spatial soil erosion NWP, UIMCL, PD, PIOV, ER, and TEMP factors indicate that these factors have significant spatial spillover or spatial lag effects on soil erosion in adjacent regions. The regression coefficients of the indirect effects of the elements all passed a significance test of at least 12%. The results are consistent, and the regression of spatial lag in terms of the explanatory variables of the soil erosion spatial model. The temporal variation of *P*-values of the driving factor index test results is shown in Figure 9.

**Figure 10:**
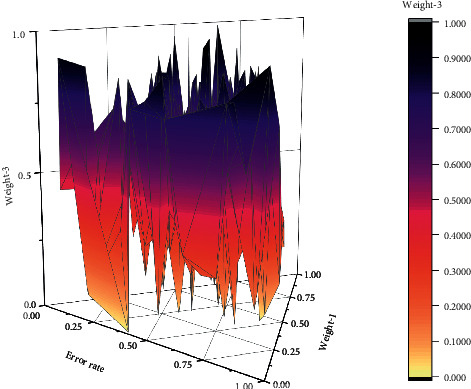
Comparison of the results of spatial and temporal characteristics of land-use change.

The model fitting error rate analysis shows that the gray neural network model plays a significant advantage in prediction. The average prediction error is below 1.75%. Still, it is widely used in combinatorial modeling, which is usually applied in the fields of industrial measurement or aviation parts height. In order to further test the rationality of space-time division of land remediation, the environmental risk index of land remediation is removed from the original index system, and an index system that only considers the urgency and suitability of land remediation is constructed. The system is determined as follows: the coordinates of the geometric center points of 140 administrative regions are used as variable inputs to construct 2   ×   140 input layer matrix as input layer parameter of SOFM neural network. The initial weights are random numbers in the interval of [0, 1], the attribute spatial weight *ωi* is 0.8, and the consequences of the criterion layer are multiplied by 0.8 to obtain the importance of the three variables in the attribute space. The significance of the three variables in the area is multiplied by 0.6 to get the weights of the attribute space, and the other parameters are all default values. The partition boundary is complete when the number of clusters is 6, as shown in Figure 10.

## 6. Conclusion

This article randomly selects several monitoring points to simulate the intermittent characteristics of the land-use change data. The interpolation of the sporadic data by applying the three prediction models concluded that the overall interpolation accuracy of the combined model on the intermittent data is higher. After experimental analysis, the improved combined model improves the accuracy of the interpolated values, especially for the data series with prominent exponential characteristics. The GWR and OLS models were also applied to regression analysis of human factors and land-use changes. By comparing the adjusted parameters of the two models, the GWR regression model has smaller parameters, and the driving factors explain the land class changes more fully. Hence, the GWR regression model is more applicable than the OLS regression model. In this study, when analyzing the drivers affecting land-use change in each subdistrict, due to the limitation of data availability, only the data that can be obtained are selected as drivers for analysis in this article, so it can only reflect the key drivers affecting land-use change to a certain extent. Since many drivers affect land-use change, more comprehensive drivers should be considered for inclusion in future studies to make the analysis more accurate and scientific.

## Data Availability

The datasets used and/or analyzed during the current study are available from the corresponding author upon reasonable request.
